# A Comparative Analysis of Psychiatric Consultations Across Emergency, Hospital, and Community Mental Health Settings

**DOI:** 10.3390/jcm15093476

**Published:** 2026-05-01

**Authors:** Rosaria Di Lorenzo, Carolina Bottone, Isabella Riguzzi, Paola Ferri, Sergio Rovesti

**Affiliations:** 1 Department of Mental Health and Drug Abuse, AUSL-Modena, 41121 Modena, Italy; c.bottone@ausl.mo.it; 2 School of Specialization in Psychiatry, University of Modena and Reggio Emilia, 41125 Modena, Italy; 3 Department of Biomedical, Metabolic and Neural Sciences, University of Modena and Reggio Emilia, 41125 Modena, Italy

**Keywords:** setting of psychiatric consultation, clinical reason for psychiatric consultation, voluntary and involuntary hospitalization, therapeutic discontinuation

## Abstract

**Background/Objectives**: A psychiatric consultation is a professional evaluation aimed at establishing a diagnosis, a prognosis, and developing a treatment plan. The objective was to assess psychiatric consultations (PCs) at the Community Mental Health Center (CMHC), Emergency Room (ER) and General Hospital (GH) to highlight differences across settings. **Methods**: With a retrospective design, we examined all PCs performed between 1 January 2024 and 31 December 2024 at the CMHC, ER and GH of Baggiovara in Modena. Descriptive statistical analysis and a multivariate logistic regression were performed. **Results**: We collected a total of 3174 PCs for 1801 patients, performed in the three settings: 52% in ER, 30% in CMHC and 18% in GH. In ER, PCs were most frequently requested for suicide risk (26%), psychomotor agitation (14%) and substance intoxication (14%). In CMHC, the most common diagnoses were depressive disorders (22%), acute anxiety (20%) and acute psychotic episodes (13%). In GH, consultations mainly addressed psychiatric symptoms associated with medical and eating disorders. The overall rate of psychiatric hospitalization after PCs was 16.2%, reaching 23.4% for consultations in ER. Discontinuation of pharmacological therapy was significantly associated with an increased risk of hospitalization (*p* < 0.001), which rose to 17% when therapy had been interrupted for more than one year. **Conclusions**: PCs at ER were the access point for most hospitalizations. Therapeutic discontinuation, acute psychosis and substance use represented the main predictors of hospitalization. Strengthening shared care pathways among CMHC, ER and GH represents an effective model of integration between hospital and community services, ensuring continuity of care.

## 1. Introduction

Psychiatric consultation is a professional evaluation aimed at establishing a diagnosis, a prognosis, and developing a treatment plan. The different contexts in which a PC is performed can be influenced by the different clinical needs of the individuals requiring counseling and, at the same time, by the different facilities available, resulting in different outcomes.

### 1.1. Urgent Psychiatric Consultations

The main settings in which a PC is required in urgency are the Emergency Room (ER) and the Community Mental Health Center (CMHC), with very different characteristics [1].

The emergency–urgency system represents a key link between hospital facilities and community services. It is organized to ensure patient care from the first moments of a critical event, including rescue, clinical stabilization, protected transport, and therapeutic continuity [2].

At the community level, General Practitioners (GPs), pediatricians, and the community emergency system play a crucial role in activating first aid pathways and ensuring the timely transfer of patients to hospital facilities when necessary.

In a hospital setting, the Emergency Room (ER) is the primary point of access for health emergencies, playing a fundamental role in defining the diagnosis and the treatment pathway [3].

The primary goal of managing psychiatric emergencies is to contain crisis, acute worsening, and re-establish a minimum level of stability favoring community or outpatient setting treatments if possible.

The term crisis is sometimes used synonymously with emergency, but the literature suggests distinguishing the two concepts. A psychiatric crisis represents a disruption of psychological equilibrium, which can appear suddenly or develop progressively, often in connection with stressful events. It does not necessarily imply an immediate threat to life but still requires targeted intervention to guide the patient toward a new equilibrium [4]. By contrast, a psychiatric emergency implies an immediate risk to the patient or others and requires urgent intervention. The American Psychiatric Association defines it as a condition characterized by an acute alteration in thinking, mood, or behavior that requires immediate intervention, based on the condition of the patient and family or social requirement [5]. It includes acute psychotic states, severe manic episodes, impaired reality testing, suicidality, and hetero-aggressive behavior [3,6].

Psychiatric emergencies constitute a substantial proportion of visits to emergency departments. In Italy, psychiatric visits to emergency departments accounted for approximately 3% of total visits in 2018, while in 2022 there were just over 547,000, with a stable but relatively increasing incidence [7].

Several studies conducted in North America have reported higher estimates, ranging from 6% to 25% of all visits to the emergency department [7,8]. In recent decades, a progressive increase in requests for urgent psychiatric intervention has been observed. This phenomenon is attributable, on the one hand, to the reduction in community resources dedicated to mental health and, on the other, to the growing prevalence of substance use disorders and psychiatric comorbidity [9]. The most common reasons for urgent psychiatric assessment include the following: acute anxiety, psychomotor agitation, acute confusion/delirium, acute psychotic episodes, depressive disorders, psychomotor retardation, self-harm and suicide attempts [9,10].

In particular, numerous international studies confirm that self-harm and/or self-destructive behaviors are the main predictors of hospitalization, along with the presence of aggressive behavior and a previous psychiatric history. In fact, suicidal behavior represents approximately 5% of all emergency department hospitalizations, with an estimated lifetime prevalence of around 13.5% in the general population [11].

A US study showed that the clinical conditions most frequently associated with psychiatric Emergency Room visits were substance abuse (27%), neurosis (26%), and psychosis (21%). Furthermore, a greater likelihood of access emerged among African American patients and those covered by the Medicaid health program, suggesting that socioeconomic and cultural variables significantly influence the use of emergency services [12].

A study conducted at the Gasthuisberg University Hospital in Leuven analyzed 1050 patients assessed by the psychiatric emergency team over a period of eight months, representing 3.5% of total emergency department visits. The majority were women (56%), with a prevalent age range between 30 and 39 years, and almost half of the subjects were unemployed. The most frequent diagnoses were mood disorders (19.7%), adjustment disorders (17.1%), and substance use disorders (14.9%). Gender differences were significant: men more frequently showed substance abuse and hostility, while women more frequently presented with suicidality (31%) and depressive symptoms (24%) [13].

In a large German cohort, suicidal ideation or behavior were confirmed as the strongest predictors of hospitalization, along with aggressive behavior, both of which were associated with higher hospitalization rates. Variables such as unemployment and housing poverty were also found to be significant in influencing the use of emergency services [14]. Another German study, using the ICD-10 classification, found that substance use disorders accounted for 37.5% of diagnoses, followed by psychosis (21.8%), neurotic disorders (18.5%), and affective disorders (11.6%). In this context, suicidality was present in 12.1% of hospitalizations and suicide attempts in 5.8% of cases [15].

A multicenter Belgian study also confirmed the centrality of anxiety and mood disorders, with a suicide rate higher than 20% [13]. In a study conducted by Kirchner and colleagues on 1733 general emergency patients, the most common diagnosis was substance use disorder, while anxiety disorders were more often associated with discharge. Among the main predictors of hospitalization were aggression, suicidal behavior, a previous psychiatric history, and inter-hospital transfer [16,17]. Overall, the data in the literature converge in indicating that affective disorders, psychosis and alcohol or drug abuse represent the most common diagnoses in psychiatric emergency services [10], highlighting that requests for urgent psychiatric intervention do not only concern psychotic or depressive crises but a broad spectrum of psychiatric conditions that include acute anxiety disorders, symptoms related to substance use, and suicidal risk situations, strongly influenced by social and environmental variables.

A historical moment that significantly influenced the demand for urgent psychiatric consultations was the COVID-19 pandemic. Indeed, the literature not only reported an increase in consultations (after an initial decline) but also an increase in complex situations, characterized by self-harm risk and acute psychosis [18,19,20,21,22]. Following the pandemic, starting in 2021, we recorded a growing increase in the total number of urgent psychiatric consultations at the CMCH, which reached its highest number in 2024 [23].

Frequent users, i.e., patients who repeatedly access emergency services for psychiatric problems, represent a significant issue. For this reason, the international literature has attempted to identify factors that increase the likelihood of repeat access to psychiatric emergency services. Specifically, from a clinical perspective, personality disorders and psychosis are strongly associated with recurrences of access, while affective disorders appear to be associated with a relatively lower risk [17].

Comorbidity with substance use disorders significantly impacts the pattern of use. In fact, patients with alcohol or cocaine dependence, combined with a comorbid psychiatric disorder, are three times more likely to access emergency services than those without comorbidities [10,17,24,25]. Some Italian studies have shown that subjects with three or more annual accesses frequently present with multiple comorbidities, particularly dual psychiatric–drug diagnosis, marginalization, or a lack of a social network [24]. The demographic and social variables most frequently associated with an increased use of ER for psychiatric consultations include middle age, male gender, economic disadvantage, unemployment, housing insecurity, and the absence of a support network [3,26,27]. In many cases, repeated use of services reflects not only clinical needs but also social vulnerability. A study conducted in Montreal highlighted that patients with frequent requests for psychiatric consultations in ER were, on average, younger, economically deprived, and had a higher prevalence of schizophrenia and comorbidities compared to those who had made isolated visits [28]. It was also noted that medical comorbidity constitutes a further significant factor. In fact, up to 50% of patients who requested a psychiatric consultation in ER also have an organic condition that could aggravate or simulate a psychiatric symptom [29,30,31]. Finally, the organizational characteristics of health systems also influence the frequency of access: in contexts where local resources are limited, the emergency department becomes the main gateway for crisis management [32,33].

### 1.2. Psychiatric Consultation in Hospital Setting

Psychiatric conditions are highly prevalent among people admitted to general medical hospitals and can affect the outcome of hospitalization [34]. The role of the Psychiatric Consultation-Liaison (C-L) is to serve as the clinical interface between general medicine and mental health, with a view towards integrating psychiatric expertise into medical care. This integrated approach to care considers the biological, psychological and social aspects of the disease, influencing its course, response to treatment and clinical outcomes [35,36].

Consultant psychiatrists contribute not only to the diagnosis and treatment of psychiatric disorders that may arise or worsen during hospitalization, but also to the recognition of psychological factors that influence the management of medical illnesses. This, in turn, promotes a person-centered culture of care [37,38].

Since their development in the 1950s and 1960s, C-L services have expanded beyond diagnostic consultation to include training, research, and interdisciplinary collaboration [39,40,41]. At present, organizational models exhibit a divergence between reactive systems predicated on on-call consultations and proactive models distinguished by regular presence in the ward and structured collaboration. The latter has been demonstrated to be associated with superior clinical outcomes, reduced length of stay, and enhanced staff satisfaction [42,43]. In recent years, a growing body of research has documented a steady increase in requests for psychiatric consultations in hospital wards, parallel to a growing awareness of the impact of mental disorders on the course of physical illnesses [44,45].

Studies conducted in several countries show that a significant percentage of patients hospitalized for medical or surgical conditions also have a psychiatric disorder, often previously undiagnosed. In fact, these disorders can affect 30% to 50% of all hospitalized patients, with significant impacts on clinical outcome, adherence to treatment, and prognosis [46,47]. However, a discrepancy remains evident between the true prevalence of psychiatric disorders in hospitalized patients and the actual frequency of consultations requested. Indeed, European data collected by the European Consultation-Liaison Workgroup (ECLW) show that only 1–1.4% of hospitalizations request a psychiatric consultation [48,49]. The causes of this underestimation of diagnoses are multiple: the lack of familiarity among non-psychiatrists with psychopathology, the tendency to interpret psychogenic symptoms from a somatic perspective, the lack of specialized resources, and the persistence of stigma surrounding mental illness [50]. This suggests that many mental health needs remain partially unexpressed or unrecognized in hospital care. Conversely, the presence of a structured liaison psychiatry service, with regular training and multidisciplinary activities, encourages an increase in requests and better management of mental health disorders [51,52].

As indicated by the extant literature, psychiatric consultations are primarily initiated by medical departments, with a notable number of such requests also originating from surgical departments and emergency departments [53].

The presence of a psychiatric disorder has been demonstrated to be associated with prolonged hospitalization, increased medical interventions and higher healthcare costs [34]. Prospective studies have demonstrated that a greater than expected proportion of patients admitted to non-psychiatric wards manifest clinically significant symptoms of anxiety, depression, cognitive dysfunction, or severe pain [46,48,49]. Individuals afflicted with such conditions have been observed to have prolonged hospitalization (40%) and elevated hospital expenses (35%) in comparison to those not afflicted with psychiatric disorders [47,54].

Among the conditions most frequently associated with a request for psychiatric consultation in medical wards are cognitive impairment, dementia and delirium [55].

Delirium constitutes a medical and psychiatric emergency, as it can represent the manifestation of potentially serious organic pathologies [56,57,58]. It is the most common psychiatric syndrome in hospitals, with a prevalence ranging from 10% to 30% in internal medicine departments and reaching 80% in intensive care units [59,60].

The extant literature also highlights that substance use disorders further complicate hospitalization, due to both the higher incidence of medical complications and the frequent co-occurrence of other psychiatric disorders, such as depression and personality disorders [61]. This is frequently associated with inadequate treatment adherence, which necessitates integrated, multidisciplinary management involving internal medicine, psychiatry and addiction services [62].

An Italian study confirms that psychiatric consultations in general hospitals frequently involve patients with affective disorders, psychosis, personality disorders and anxiety-related conditions [63].

An observational study conducted at Perugia General Hospital demonstrated that over half of the requests for psychiatric consultation originated from medical wards (53.1%), followed by specialist wards (38.1%) and surgical wards (8.8%). The primary reasons for referral PCs included anxiety disorders (18.9%), depressive symptoms (18.2%), confusion states (13.4%), somatic symptoms not explained by organic causes (11.2%), risk or attempted suicide (11.2%), and psychomotor agitation (10.9%), as well as a positive psychiatric history [64].

It is important to note that delays in referral to psychiatric services are associated with prolonged hospitalization, with each day of delay potentially extending the length of stay by approximately two days [65]. This confirms the effectiveness of proactive consultation models, which include the psychiatrist’s regular presence on the wards and participation in discharge planning.

Studies comparing psychiatric consultations in Emergency Rooms, Community Mental Health Centers, and general hospitals are scarce in the literature, with limited knowledge of context-specific demand patterns and outcomes.

### 1.3. The Objectives of This Study

The primary objective of this study is to evaluate the frequency, diagnoses, interventions, and outcomes of urgent psychiatric consultations performed at a Mental Health Center (CMHC) of a community service, at an Emergency Room (ER) and at medical wards of a General Hospital (GH) during a period of one year. The secondary objective of this study is to highlight the different patterns of clinical requests and therapeutic care interventions based on the three different settings.

## 2. Materials and Methods

### 2.1. Study Design/Methodology

This observational, retrospective, and single-center study was conducted in the Department of Mental Health and Drug Abuse of Modena Centre.

The organization of mental health services in the province of Modena is part of the Emilia-Romagna regional model, based on the integration of community and hospital services within a single departmental network. This network is coordinated by the Department of Mental Health and Drug Abuse (DSM-DA) of the AUSL-Modena, which is responsible for ensuring continuity of care, timely interventions, and appropriate treatment for adult and minor populations with psychiatric disorders [66]. The regional model, developed from the principles of Law 180/1978 and further defined through the Regional Guidelines, is based on the cornerstones of community psychiatry. The goal is to promote care in the community, prevent institutionalization, and ensure a continuum of care between outpatient services and residential/hospital admissions [67].

Our local Community Mental Health Center (CMHC) is divided into three main services, which are the community-based facilities responsible for adult psychiatric care and are the primary points of access for the population. The CMHC guarantees opening hours (from 8:00 a.m. to 8:00 p.m. on weekdays and until 2:00 p.m. on Saturdays) for ensuring psychiatric consultations (PCs) at the CMHC in cases of psychotic breakdown, psychomotor agitation, suicidal risk, or acute behavioral disturbances [64]. The CMHC relates to the Service for Psychiatric Diagnosis and Care (SPDC) in the General Hospital in Baggiovara (Modena), which represents the hospital’s referral center for psychiatric consultations in the ER and in the medical wards of the General Hospital where it is located, providing continuity of care 24 h a day. During nights and holidays, psychiatric consultations (PCs) are available 24 h a day in the ER of the GH in Baggiovara (Modena), where a psychiatrist is on duty as ER and GH consultant and as physician of the SPDC ward. The SPDC is a ward where voluntary and involuntary hospitalizations, according to 180/78 and 833/78 Laws, are carried out (catchment area of 700,000 people).

At the GH in Baggiovara, psychiatric service provides specialist consultations, both in the emergency department and in the internal medicine, surgical, and specialist departments. Psychiatric consultations in the emergency department are provided in cases of psychiatric emergency, such as psychomotor agitation, suicidal ideation, substance abuse, acute psychotic symptoms, or confusion states. In these situations, the on-call psychiatrist intervenes promptly to perform a clinical assessment and determine the most appropriate outcome: discharge with referral to the Mental Health Center (CMHC), temporary observation, or admission to psychiatric wards.

Psychiatric consultations in hospital wards are requested for patients hospitalized for medical conditions who present psychiatric symptoms during their stay. In these cases, the consultation supports pharmacological management, suicide risk assessment, the doctor–patient relationship, and planning for continuity of care after discharge. This activity falls within the framework of consultation and liaison psychiatry, which promotes integration between medical and mental health disciplines, reducing the gap between general medicine and psychiatry and improving the overall quality of care [68].

#### Sample and Variables

We collected all PCs required for patients aged older than 14 years at CMHCs, the ER and GH in Baggiovara (Modena) between 1 January 2024 and 31 December 2024. Psychiatric consultations were extracted consecutively in chronological order, including all consultations recorded in the database during the study observation period. No additional selection criteria were applied.

In the patient-level analyses, only the first psychiatric consultation for each patient during the study period was considered. In contrast, in the consultation-level analyses, all psychiatric consultations were included, and repeated consultations from the same patient were treated as independent observations.

We analyzed the following variables:

- Patient socio-demographic data (age, sex, nationality and residence).
- Location of consultation: CMHC, ER and GH.
- Work shift during which consultation was performed: daytime, weekdays/holidays, nighttime.
- Clinical reason for PCs. The categories used to describe the clinical reasons for consultation (e.g., anxiety states, agitation, suicide attempts) represent groupings of the most frequently observed clinical presentations during psychiatric consultations.
- Referral to PCs: spontaneous, unaccompanied/accompanied, General Practitioner (GP), ER physicians, other services, law enforcement agencies, or by other specialists.
- Previous care at CMHC or other service or specialist.
- Previous psychopharmacological therapy.
- Therapeutic adherence: Regular intake of therapy, therapy interruption for less than three months, for three months to one year, and for more than one year. Therapeutic adherence was assessed based on information obtained during the psychiatric consultation, including both patient self-report and review of the available medical records.
- Interventions performed during PCs: Individual/family interview, administration/prescription of psychopharmacological therapy, request for additional tests (blood tests/urine toxicology/electrocardiogram/specialist consultation).
- PC outcome; hospitalization, referral to CMHCs and/or other community services, referral to GP, request for psychiatric re-evaluation and/or other specialist consultation, voluntary or involuntary hospitalization.
- Psychiatric diagnosis, presence of organic comorbidity and/or concomitant substance use according to ICD-9-CM classification system, which is routinely used in our clinical setting.
- Number of consultations per patient during the observation period.

### 2.2. Statistical Analysis

Descriptive statistical analysis was performed on the demographic and clinical data:

- For continuous variables: mean (m) ± standard deviation (SD), *t*-test after analyzing the normality of the distribution with the Shapiro–Wilk test (in case of non-normal distribution, the Kruskal–Wallis test was applied).
- For categorical variables: percentage values and Pearson χ^2^ test.

A multivariate logistic regression was performed between the dependent variable “Hospitalization” (1 = hospitalization, 0 = no hospitalization) and the other selected variables, using the stepwise regression method, to highlight which variables influence the need for hospitalization. The statistical probability level was set at *p* < 0.05.

All data were analyzed using STATA Version 12 software.

### 2.3. Privacy and Ethical Considerations

This study was conducted in accordance with the Declaration of Helsinki and approved by the Ethics Committee of the Emilia Nord Area (Prot. AOU 0014271/25 of 19 May 2025) and authorized by the Modena Local Health Authority (Determination No. 1511 of 30 May 2025).

The study data were collected anonymously, assigning each selected patient a progressive numerical code, and analyzed using statistical methods to obtain the information required for the research. Access to the data was granted to the study manager and his/her collaborators, who were bound by the obligation of confidentiality and data processing in accordance with current regulations. Consultation data were obtained through the computerized system used in SPDC and CMHC.

This study is reported in accordance with the STROBE (Strengthening the Reporting of Observational Studies in Epidemiology) guidelines.

## 3. Results

### The Sample of PCs in CMHC, ER and GH

The sample analyzed includes a total of 3174 PCs across the three different settings examined: 1651 (52%) in the ER, 573 (18%) in the wards of GH, and 950 (30%) at the CMHCs. The overall sample of patients requiring consultations consisted of 1801 individuals, of whom 1084 (60%) were assessed in the ER, 282 (16%) in the GH, and 435 (24%) at the CMHC. The number of PCs per patient was statistically signifcantly different across the three settings (Pearson chi2 = 32.60, *p* = 0.000): in the ER, 957 (88%) of patients required only one PC (SR ≥ 2, *p* < 0.05) vs. 127 (12%) who required more than one PC during the study period; in the GH, 211 (75%) required only one PC; whereas 71 (25%) more than one PC (SR ≥ 2, *p* < 0.05); and in the CMHC, 366 (84%) required only one PC and 69 (16%) more than one PC. The gender distribution of consultation subjects appears substantially balanced, 49% male and 51% female, with no statistically significant differences across the three settings. Conversely, the mean age across the three settings shows significant variations (χ^2^ = 164.199, *p* = 0.0001, Kruskal–Wallis, given that the normality test showed a *p* = 0.000): patients in the wards are on average older (57 years), while in the ER and CMHC the mean age is 40.5 and 44.6 years, respectively, a statistically significant difference.

In Table 1, the residence of people requesting a psychiatric consultation is shown: residence in Modena and province was high overall (90%), reaching 98% in the CMHC (*p* < 0.05). In the ER, a higher proportion of non-residents was observed: Italian citizens in 11% of cases (RS ≥ 2, *p* < 0.05) and people from non-European countries in 4% (RS ≥ 2, *p* < 0.05), with a statistically significant difference (Pearson χ^2^ = 101.96, *p* = 0.000).

**Figure 1:**
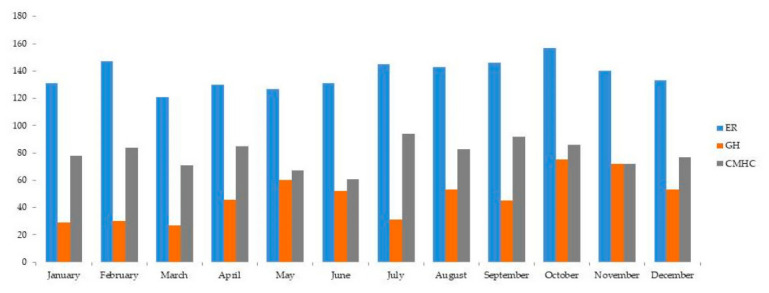
Monthly distribution of PCs in ER, GH and CMCH.

The seasonal trend in consultations, shown in Figure 1, presented a slight increase in autumn (27.8%), followed by summer (25%), winter (24%), and spring (23.2%). The only statistically significant variation was found in the PCs in the GH, where consultations increased in May, October, and November (RS ≥ 2, *p* < 0.05; Pearson χ^2^ = 56.68, *p* = 0.000).

Monthly trends showed a higher concentration of consultations in summer, particularly between July and September, and a slight decrease in autumn. In January and April, PCs showed statistically significant differences compared to other periods of the year (Pearson χ^2^ = 54.133; *p* = 0.012; RS ≥ 2, *p* < 0.05).

The distribution of PC referrals showed statistically significant differences (Pearson χ^2^ = 2.5 × 10^3^; *p* < 0.001) among the three settings, as shown in Table 1. In the ER, unaccompanied spontaneous referral prevailed (40%); in the GH, most PCs were requested directly by the staff medical wards (72%) or by consultant psychiatrists for clinical re-assessments (26%); in the CMHCs, consistently with their function of providing continuity of care, spontaneous requests for PCs prevailed (61%), followed by those sent by other community services (14%) and General Practitioners (5%).

The distribution of PCs by work shift (Table 1) also differed across the three settings (Pearson χ^2^ = 762.82; *p* = 0.000): in CMHCs, PCs take place exclusively during weekdays (100%); in the ER, most PCs are carried out at night (41%) and on holidays (10%); and in the GH approximately half of consultations (44%) take place at night, suggesting urgent modality in all three settings.

In CMHCs, almost all patients (91%) were already being cared for by the CMHC, whereas in the GH, the percentage drops to 63% and in the ER to 72%, with a statistically significant difference (Pearson χ^2^ = 181.11, *p* = 0.000). Therefore, patients not in care were more represented in hospital settings, with 26% in the ER and 35% in the GH wards (Table 1).

Regarding the hospital departments which requested a PC, the prevalent medical areas (44.8%) were Internal and Metabolic Medicine, Gastroenterology, Geriatrics, Cardiology, and Neurology, followed by emergency and urgent care areas (36.8%), such as Emergency Medicine, Intensive Critical Care, and Anesthesia and Resuscitation/Intensive Care; surgical areas (Gynecology and Obstetrics, Orthopedics, General Surgery) represented 10.7% of PC requests, followed by Rehabilitation areas (7.2%), and finally by Oncology (0.5%).

The clinical characteristics of PCs, reported in Table 2, highlight further differences between the contexts examined. The different ways in which patients took psychopharmacological therapy differed statistically significantly in the three settings (Pearson χ^2^ = 421.60, *p* = 0.000): in the CMHC, most patients were undergoing oral therapy (73%), with a significant proportion of long-acting (LAI) therapy combined (13%) and alone (4%); in the GH, parenteral therapies were more frequent; whereas in the ER, approximately 31% of patients were not receiving pharmacological treatment at the time of PC evaluation (Table 2).

Therapeutic adherence was generally good (89%) but differed significantly across settings (Pearson χ^2^ = 75.31, *p* = 0.000): it was higher in the GH (95%) and slightly lower in the CMHC (90%), where therapy interruptions of more than 3 months (9% of cases) were statistically significant. In the ER, short (<3 months = 7%) or prolonged (>1 year = 2%) therapy discontinuations were observed in a statistically significant way (Table 2).

The presence of medical comorbidities was also analyzed in the clinical characteristics, which are more frequent in the GH (42%), compared to 14% in the ER and 5% in the CMHC (Table 2).

The clinical reasons for consultations, reported in Table 3, statistically significantly differed across the three clinical contexts (Pearson χ^2^ = 951.90, *p* = 0.000): in the ER, PCs were most frequently requested for self-harm risk or suicide attempt (26%), followed by agitation/aggressive behavior (14%) and substance/medication intoxication or withdrawal (14%). In the GH, PCs were more frequently requested for mental disorders secondary to medical disorders, present in 16% of PCs, followed by eating disorders (8%) and monitoring psychopharmacological therapy (4%). Finally, PCs in the CMHC were primarily requested for depressive symptoms (22%), acute anxiety states (20%), and acute psychosis (13%) in a statistically significant way.

The clinical interventions performed during psychiatric consultations (Table 4) showed statistically significant differences (Pearson χ^2^ = 399.01, *p* = 0.000). Specifically, in the emergency department, individual or family interviews prevailed (34%), followed by therapy administration (22%) and combined interventions of consultation, therapy administration, and therapeutic prescription (11%). In the GH, there was a clear prevalence of consultations which modified and monitored psychopharmacological therapy prescription (54%) and requested hematological tests and non-psychiatric consultations (4%). In the CMHC, a combination of interview and prescription home therapy (39%) and the activation of compulsory health assessment (3%) were statistically significantly more frequent.

Statistically significant differences were reported in the outcomes of PCs in the three different settings (Pearson χ^2^ = 1.5 × 10^0.3^, *p* = 0.000) (Table 5): in the ER, over half of patients (51%) were discharged and referred to community services, and unlike in other consultation settings, 18% were voluntarily admitted, 4% were admitted to a non-psychiatric setting, and 3% were discharged and sent to a private specialist or to prison; in the GH, the most frequent outcome was the reassessment of mental conditions in the continuation of hospitalization (69%); whereas in the CMHC, the most frequent outcome was referral to community services (70%), followed by scheduled hospital admission (5%) and out-of-hospital compulsory treatment, according to Laws 180/78 and 833/78 (1%). The percentage of voluntary and involuntary hospitalizations to a psychiatric ward was 13% and 2%, respectively, with a statistically significant difference between the clinical settings where a PC was performed: 18% and 3% from the ER, 7% and 1% from the GH, 6% and 2% from the CMHC.

**Figure 2:**
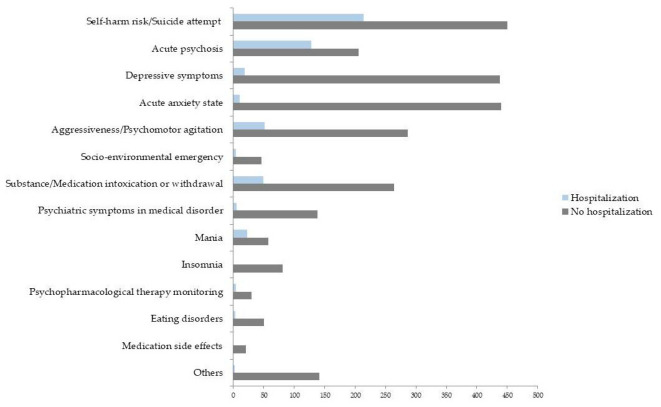
Hospitalization as outcome of PCs by clinical reason.

Regarding the outcome hospitalization of PCs grouped by clinical reason, as reported in Figure 2, we observed that patients at risk of self-harm (32%) and in acute psychosis (38%) had the highest hospitalization rates in a statistically significant way (Pearson χ^2^ = 436.77, *p* = 0.000).

In particular, regarding outcomes of PCs in the ER, GH, and CMHC by clinical reason, we reported that mania and aggressiveness/agitation were the PC outcomes statistically significantly related to involuntary hospitalization, whereas self-harm risk/suicide attempt was related to voluntary hospitalization and acute psychosis both to voluntary and involuntary hospitalization; conversely, other clinical reasons, such as depressive symptoms, acute anxiety state, insomnia, psycho-pharmacotherapy monitoring and medication side effects, were statistically significantly related to referral to CMHC/other community services (Pearson’s χ^2^ test. χ^2^ = 629.958, *p* < 0.001; Table 6).

In Table 7, the relationship between clinical reason for PCs and therapeutic adherence is reported: self-harm risk/suicide attempt (22%) and acute anxiety states (15%) prevailed among adherent patients, whereas among those who discontinued therapy for a long time, the most frequent clinical reasons were acute psychosis (32%) and substance/medication intoxication or withdrawal (22%) (Pearson χ^2^ = 164.19, *p* = 0.000).

The relationship between PC outcomes and therapeutic adherence (Table 8) highlighted statistically significant differences (Pearson χ^2^ = 263.97, *p* = 0.000): subjects with good adherence were predominantly referred to community services (50%), differing from other subjects with treatment discontinuation who were voluntary and involuntary hospitalized. Among those with therapeutic discontinuation of more than 3 months and less than 1 year, voluntary hospitalization (27%), involuntary hospitalization (6%), and out-of-hospital involuntary treatment (2%) were more frequent. Among patients who had discontinued therapy for more than 1 year, involuntary hospitalizations (17%) and requests for consultation with a non-psychiatric specialist (4%) prevailed.

Regarding substance use, we reported an increase in multiple substances and cannabinoid use among subjects with a therapeutic discontinuation of less than 3 months and more than 1 year, whereas among patients who were adherent to psychopharmacological therapy, the absence of substance use prevailed in a statistically significant way (Pearson χ^2^ = 68.97; *p* = 0.000). Moreover, we observed that 87% of patients assessed in CMCH did not use any substances, whereas 10% of those assessed in ER used alcohol, 11% used multiple substances, and 5% used cannabinoids (Pearson χ^2^ = 54.53, *p* = 0.000).

Our model of multivariate logistic regression (Table 9) between hospitalization (dependent variable) and selected variables (independent variables) highlighted that acute psychosis and socio-environmental emergencies were predictive of hospitalization, whereas depressive symptoms, acute anxiety state, eating disorders, substance/medication intoxication or withdrawal, psychiatric symptoms in medical disorder, and psychopharmacological treatment monitoring were protective for hospitalization. Furthermore, all types of interventions performed during PCs were negatively and statistically significantly associated with hospitalization, proving to be protective for hospitalization.

## 4. Discussion

This study analyzed the PCs carried out during 2024 in the Emergency Room and in the medical wards of the General Hospital of Baggiovara (Modena), as well as in the Community Mental Health Center of Modena (Italy) in comparison.

The large sample size and the variety of clinical settings allowed us to highlight the clinical needs of the population who required a psychiatric consultation in a real-world setting, providing a faithful report of the organization of PCs in the three settings.

Psychiatric consultation in ER is a specialized medical assessment performed by a psychiatrist for acute clinical symptoms requiring mental health expertise for evaluating a diagnosis, a prognosis, and performing a planned treatment. Psychiatric consultations in medical hospital wards are a key intervention that favors the integration of the psychological and somatic dimensions of patients, promoting continuity of care. Psychiatric consultations in the CMHC represent the first point of access in a community setting for acute psychiatric conditions as well as the continuation of community psychiatric care for people already treated at a mental health service.

Our results show that over half (52%) of PCs were performed in the ER, 30% in the CMHC, and 18% in hospital wards of the GH in the study period. This distribution confirms the central role of the emergency department as the main point of access to urgent psychiatric consultations, which indicates the ER as a key hub for intercepting mental health crises, consistently with the literature [2,8]. We confirm that the ER was the main point of access for people not yet treated at a CMHC and/or characterized by social fragility, lack of a support network, or marginalization. This finding is consistent with the literature, which describes the emergency department as a true “safety net” for the most vulnerable individuals [12,26]. At the same time, the high number of consultations performed in the CMHC highlights the ability of community services to promptly respond to emergencies, reducing and preventing hospitalizations. This result suggests the efficiency of the community model in psychiatry, which relies on the connection between community services and the hospital to provide extensive and continuous care [67].

In the CMHC, however, urgent consultations primarily concerned patients already treated by the service, who were experiencing an exacerbation or worsening of psychiatric disorders, whereas psychiatric consultations in hospital wards were focused on specialized support for patients hospitalized with medical illnesses. This distinction is consistent with the liaison psychiatry model described in the literature, which emphasizes the importance of adapting the intervention to the clinical and care context [46,52].

From a demographic perspective, significant differences emerge between clinical settings. In hospital wards, patients were older than those who required a PC in the ER and CMHC, where a population of young and/or middle-aged adults prevailed. Almost all patients came from the catchment area of the CMHC and GH, with differences between the three clinical settings: the ER welcomed a higher proportion of non-residents and/or foreign people, particularly non-Europeans, confirming its role as the first point of contact for the most vulnerable social groups or those not yet integrated into health and/or social services, confirming an essential role of the ER in filtering emerging needs and intercepting clinical conditions of distress that would otherwise risk being excluded from structured care pathways.

We also observed significant differences in the incidence of psychiatric disorders across the three settings: a higher incidence of delirium and cognitive disorders was noted in the GH, whereas in both the ER and CMHC, mood disorders, substance abuse, and self-harm risk were more frequent, in line with literature [14]. Furthermore, a high presence of medical comorbidity (42%) was observed among people hospitalized in the GH, confirming the crucial role of liaison psychiatry in the management of complex patients, in which the psychopathological component significantly impacts the clinical course and length of hospital stay [47,52]. Consistent with the literature, most psychiatric consultations were requested by medicine wards, followed by surgery areas [52].

Regarding the access to psychiatric consultations, our study underscores results consistent with those highlighted by other authors, since the majority of PCs in the ER were required by physicians of the emergency department or self-referred by patients accompanied by a family member or caregiver [13], whereas most PCs in the CMHC were self-referred by unaccompanied patients or referred from other community services; finally, PCs required for a re-evaluation were often required in the GH.

Furthermore, consistent with the literature, the data analysis revealed significant differences in the reasons for requesting consultations, based on the three different clinical settings. In particular, the risk of self-harm risk or suicide attempts (26%), psychomotor agitation (14%), and substance/medication intoxication or withdrawal (14%), i.e., acute and serious clinical conditions that may require hospitalization, were prevalent in the ER, as reported in the literature [14,16,17]. Mental disorders secondary to medical conditions and eating disorders were more frequently diagnosed in people hospitalized in a GH, whereas depressive disorders, acute anxiety states, and psychotic disorders were prevalent in the CMHC. Suicidal behaviors and acute psychotic episodes represented clinical situations that, especially in the presence of therapeutic discontinuity or social isolation, required hospitalization, confirming the literature [14,47]. Regarding therapeutic non-adherence, although our results do not establish a causal relationship, they clearly indicate that therapy discontinuation in both the short and long term is associated with an increased risk of hospitalization, whether voluntary or involuntary. This finding underscores the importance of treatment continuity in reducing hospitalization, beyond other clinical and social factors [23,69,70].

Toxicological comorbidity emerges as a significant cross-sectional finding since over a quarter of patients assessed in the ER had a substance use disorder, a finding consistent with the high percentage of dual diagnosis reported in the literature [9,28].

In our sample, we did not report the so-called “frequent users” of ER or patients with high consumption of health and social resources [32], because only 12% of patients in the ER and 16% in the CMHC required more than one PC during the study period (the higher number of patients who required more than one PC in the GH can be interpreted as the need for repetitive evaluations in case of complex comorbidities).

We reported a difference in hospitalization rate based on the setting where a PC was carried out: 23.4% of patients assessed in the ER were admitted to the hospital, compared to 8.6% of people evaluated in the GH and 8.5% in the CMHC. This confirms that the ER is not only the main point of access for psychiatric emergencies but also the setting with the highest hospitalization rate, reflecting the greater clinical severity of the cases referred to it and, at the same time, a stronger patient demand for hospitalization, which the consultant is unlikely to ignore or deny.

The differences observed across CMHC, ER, and GH settings may reflect the distinct organizational roles and clinical functions of these services within the mental health care system. The high proportion of consultations in the ER, particularly for suicide risk, psychomotor agitation, and substance intoxication, suggests that this setting primarily manages acute psychiatric crises and represents a key access point for more severe clinical presentations. In contrast, the CMHC appears to address a broader range of non-emergency conditions, including depressive and anxiety disorders, which are more consistent with ongoing outpatient care and longitudinal management. Consultations in the GH were mainly related to psychiatric symptoms associated with medical comorbidities, supporting the role of consultation-liaison psychiatry in integrated hospital care.

The multivariate analysis confirms our findings, highlighting how the risk of hospitalization increases in the event of discontinuation of pharmacological therapy as well as in the presence of serious clinical conditions such as acute psychosis and comorbidity with substance use, whereas many other clinical reasons for PCs did not represent risk of psychiatric hospitalization. In particular, the association between therapeutic discontinuation and increased risk of hospitalization highlights the potential role of treatment adherence in the clinical trajectory of patients, although causal inferences cannot be drawn due to the observational design. Similarly, the association of acute psychosis and substance use with hospitalization is consistent with their clinical severity and the need for more intensive management. Conversely, PCs performed at the CMHC, which led to subsequent referrals for follow-up consultations at community services, may represent a protective factor against hospitalization, ensuring continuity of care, as do all interventions (interview, medication prescription, prescription home therapy, request for laboratory tests and/or non-psychiatric consultation) performed during psychiatric consultations. This result underscores the protective function of both community service care and clinical activities aimed at meeting patients’ needs, reducing the risk of hospitalization.

Another point concerns the distinction between voluntary and involuntary hospitalizations: most hospitalizations were voluntary (86%), whereas involuntary hospitalizations (14%) represented PC outcomes for people with prolonged therapeutic discontinuation, acute psychosis, mania and aggressiveness/agitation, in line with the 2020 APA and 2019 Ministry of Health recommendations [5,7], which show how these clinical conditions represent risk factors for both compulsory treatments and potential for injury to patients and staff in the ER due to the escalation of aggressive and violent behavior [71]. For these reasons, the ER, boarding people with psychiatric disorders, needs careful consideration of management plans to mitigate patient safety events, such as Project BETA (Best Practices in the Evaluation and Treatment of Agitation), which consists of the compilation of the best evidence and consensus recommendations in behavioral emergencies [72].

Therapeutic adherence was high (89%) in our sample of people but significantly lower among people requiring PC in the ER instead of the GH and CMHC. The correlation between adherence and outcome is clear: patients who had suspended treatment experienced an increase in hospitalizations, especially in involuntary ones, whereas patients with good therapeutic continuity were more likely to be referred to community services. These results confirm that pharmacological discontinuity is one of the main predictors of relapse and re-hospitalization while therapeutic adherence is a key indicator of good prognostic outcome and clinical stabilization [5,17].

Patients already receiving care from community services show better levels of adherence, a lower likelihood of hospitalization, and greater clinical stability. This highlights the fundamental role of community psychiatry and integrated care in the prevention of acute conditions, implementing psycho-educational interventions, regular monitoring, and the use of long-term therapies, which represent effective strategies for promoting continuity of care according to the PERSEO survey [63] and the study by De Giorgio et al. [64]. These findings, taken together, suggest that differences across settings may be influenced not only by patient characteristics but also by the organization and accessibility of services, as well as by patterns of care continuity. Therefore, the results support the relevance of integrated care pathways linking CMHC, ER, and GH to facilitate appropriate management across different levels of care.

Overall, the results of this study confirm the validity of hospital–community integration, based on ongoing collaboration between SPDC, ER, and CMHC. Extensive psychiatric availability throughout the day, organizational flexibility, and continuity of care permit effective crisis management and reduce hospitalization, consistent with the regional recommendations [37].

The seasonal pattern, with an increase in PCs during the summer months in hospital settings but not in the other clinical contexts, also suggests the potential influence of environmental and social factors on psychopathological exacerbations in medical comorbidity, offering useful insights for seasonal resource planning [73,74,75].

In light of our results, we can conclude that, as previous studies have shown, the organization of healthcare systems significantly influences the pattern and frequency of psychiatric consultations. In Italy, territorial differences in service organization and the balance between hospital-based and community care have been associated with variations in emergency psychiatric presentations and consultation pathways [76]. In particular, Italy has one of the lowest rates of psychiatric inpatient beds in Europe (approximately 9–10 beds per 100,000 inhabitants), markedly lower than other high-income countries [77]. This reduced inpatient capacity, largely related to the community-based model introduced after the psychiatric reform, may contribute to increased pressure on emergency services and influence pathways to care [78]. Furthermore, international evidence suggests that the current availability of psychiatric beds in several countries, including Italy, remains below the estimated minimum required to adequately meet population needs [79]. Moreover, changes in healthcare delivery, such as those observed during the COVID-19 pandemic, have demonstrated how modifications in service availability can directly impact both the volume and nature of psychiatric consultations [19,20,22,80]. For instance, Italian studies have reported significant changes in consultation rates and clinical presentations in emergency settings before and after the pandemic, highlighting the role of system-level factors in shaping access to care. Similar findings have been reported internationally, where the structure and integration of mental health services within emergency and general hospital settings, such as consultation-liaison models or collaborative care, play a crucial role in influencing access, length of stay, and clinical outcomes. In particular, a recent systematic review conducted on 18 studies showed that integrated care models shorten hospital stays, enhance patient outcomes, and expand access to psychiatric treatments, particularly in remote locations [81].

### Limitations and Advantages of the Study

The main limitation of this study is its retrospective, single-center design, which does not allow us to establish causal relationships between variables. Moreover, its monocentric design limits the generalizability of the findings to other regional or national contexts. A further critical issue is the lack of longitudinal post-discharge follow-up, which would have allowed us to assess treatment adherence and medium-term outcomes in terms of relapses and re-hospitalizations over time. Regarding the statistical analysis, the use of a stepwise regression model may introduce potential bias or model overfitting; however, it was employed for exploratory purposes. Furthermore, some clinical variables, such as symptom severity or duration, were not consistently documented in computerized records, and psychiatric diagnoses may have varied across clinicians, thereby limiting the level of detail in the analysis. Finally, the potential clustering effect due to repeated PCs within the same patient was not fully accounted for and may have influenced the estimates.

This study’s strengths include its large sample size, its representativeness of the three main care settings, and the numerous variables considered, which allow us to gain a comprehensive understanding of the phenomenon. The analysis of data from the ER, GH and the CMHC reports an organizational model consistent with the most recent national and regional guidelines.

## 5. Conclusions

This study highlights the activity and organization of psychiatric consultations in three different clinical settings and, concomitantly, underscores the clinical characteristics and needs of people requiring PC and the functioning of an integrated mental health service network.

Our findings highlight that the ER is the primary point of access for psychiatric emergencies, with a prevalence of severe conditions characterized by suicidal risk, psychomotor agitation, intoxication, and acute psychosis. In CMHC, however, most PCs concern individuals already in care with exacerbation of depressive or anxiety disorders. In hospital wards, PCs are requested for liaison for patients with medical comorbidities, confirming the importance of consultation psychiatry in the integrated management of complex patients. These observations indicate different populations of patients with diverse clinical demands and needs, requiring specific and personalized, though integrated and continuous, interventions. Hospitalization occurred in 16% of all consultations, mostly on a voluntary basis, and especially when a PC was carried out in the ER. This reflects the severity of the cases presented to the Emergency Room and/or the request for hospitalization by the patients themselves, who go to the hospital in an emergency. Our results also highlight that the outcome of most PCs is not represented by hospitalization as therapeutic resource but is differentiated and diversified based on severity and acuity of clinical conditions, favoring the continuity of care at community settings when it is possible. Our analysis highlights that therapeutic discontinuation, even for a short period, can represent a risk factor for hospitalization, along with acute psychosis.

Our results suggest several relevant implications for service organization and crisis management. First, the high proportion of psychiatric consultations in the ER prescribing hospitalizations indicates that ER services represent a key access point for acute psychiatric care. This highlights the need to strengthen emergency psychiatric resources, including staff training in the management of suicide risk, psychomotor agitation, and substance-related conditions. Second, the strong association between therapeutic discontinuation and hospitalization underscores the importance of continuity of care within community services. Enhancing follow-up strategies, adherence monitoring, and early intervention programs at the CMHC level may help prevent acute psychosis and reduce emergency service utilization. Finally, the differences observed across CMHC, ER, and General Hospital (GH) settings support the need for improved integration between hospital and community mental health services. The implementation of shared care pathways, structured referral systems, and consultation-liaison models may facilitate more timely and appropriate management of psychiatric patients, ultimately reducing avoidable hospitalizations and improving overall patient outcomes.

In light of our findings, which confirm the integration between community service and hospital ER, we emphasize that constant cooperation between the various levels of care, combined with shared protocols and multidisciplinary teams, allows us to maintain high standards of quality care, effectively responding to the needs of those accessing psychiatric consultations. Overall, our results confirm the validity of the integrated mental health model, based on timely intervention, continuity of care, and close coordination across the various levels of care.

## Figures and Tables

**Table 1:** Organizational characteristics of PCs in ER, GH and CMHC.

Variables	PCs in ER (n = 1651)	PCs in GH (n = 573)	PCs in CMHC (n = 950)	Total PCs (n = 3174)
**Residence, n (%)**				
Modena and Province	1405 (85%)	503 (88%)	927 (98%) *	2839 (90%)
Italy	186 (11%) *	50 (9%)	21 (2%)	257 (8%)
Europe (excluding Italy)	10 (1%)	5 (1%)	0	15 (0%)
Non-European Countries	44 (4%) *	15 (3%)	1 (0%)	60 (2%)
**Referring Source, n (%)**				
Self-referred, unaccompanied	666 (40%)	3 (1%)	577 (61%) *	1248 (39%)
Other hospital ward	69 (4%)	412 (72%) *	14 (1%)	495 (16%)
Emergency department	369 (22%) *	2 (0%)	14 (1%)	385 (12%)
General Practitioner (GP)	18 (1%)	0	48 (5%)*	66 (2%)
CMHC/Substance use service/Other services	96 (6%)	2 (0%)	134 (14%) *	234 (7%)
Prison	4 (0%)	1 (0%)	2 (0%)	7 (0%)
Compulsory health assessment **	156 (9%) *	1 (0%)	25 (3%)	182 (6%)
Psychiatric re-evaluation	100 (6%)	151 (26%) *	93 (10%)	344 (11%)
Self-referred, accompanied	173 (10%) *	1 (0%)	42 (4%)	216 (7%)
**Work shift, n (%)**				
Weekday daytime	801 (49%)	267 (47%)	950 (100%) *	2018 (64%)
Nighttime	668 (41%) *	253 (44%) *	0	923 (29%)
Holiday daytime	171 (10%) *	52 (9%)	0	225 (7%)
**Under care of community services, n (%)**				
Not under care	433 (26%) *	201 (35%) *	77 (8%)	711 (23%)
Under care	1194 (72%)	362 (63%)	863 (91%) *	2419 (77%)
Unknown	24 (1%)	10 (2%)	10 (1%)	44 (1%)

Statistical analysis: Pearson’s χ^2^ test. Residence: χ^2^ = 101.96, *p* = 0.000. Referring source: χ^2^ = 2500.00, *p* < 0.001. Shift: χ^2^ = 762.82, *p* < 0.001. Under care of services: χ^2^ = 181.11, *p* < 0.001. * Standardized Residuals (SR) ≥ 2, *p* < 0.05. ** According to Laws 180/78 and 833/78.

**Table 2:** Clinical characteristics of people requiring PC in ER, GH and CMHC.

Variables	PCs in ER (n = 1651)	PCs in GH (n = 573)	PCs in CMHC (n = 950)	Total PCs (n = 3174)
**Previous Psychopharmacological Treatment, n (%)**				
No treatment	519 (31%) *	92 (16%)	85 (9%)	696 (22%)
Oral therapy	999 (61%)	399 (70%)	696 (73%) *	2094 (66%)
Long-acting injectable (LAI)	20 (1%)	9 (2%)	41 (4%) *	70 (2%)
Oral + LAI therapy	79 (5%)	25 (4%)	126 (13%) *	230 (7%)
Intravenous/Intramuscular (IV/IM)	6 (0%)	30 (5%)*	0	36 (1%)
Oral + IV/IM therapy	2 (0%)	15 (3%)*	0	17 (1%)
Unknown	26 (2%)	3 (1%)	2 (0%)	31 (1%)
**Therapeutic Adherence, n (%)**				
Good adherence	956 (85%)	451 (95%) *	774 (90%)	2181 (89%)
Discontinuation < 3 months to 1 year	74 (7%)	19 (4%)	74 (9%)*	167 (7%)
Discontinuation < 1 year	75 (7%) *	5 (1%)	13 (2%)	93 (4%)
Discontinuation > 1 year	20 (2%) *	1 (0%)	2 (0%)	23 (1%)
**Medical comorbidity, n (%)**				
Absent	1413 (86%) *	332 (58%)	901 (95%)	2646 (83%)
Present	238 (14%)	241 (42%) *	49 (5%)	528 (17%)

Statistical analysis: Pearson’s χ^2^ test. Previous psychopharmacological treatment: χ^2^ = 421.60, *p* < 0.001. Treatment adherence: χ^2^ = 75.31, *p* < 0.001. Comorbidity: χ^2^ = 363.18, *p* < 0.001. * Standardized Residuals (SR) ≥ 2, *p* < 0.05.

**Table 3:** Clinical reasons for PCs in ER, GH, and CMHC.

Variables	PCs in ER (n = 1651)	PCs in GH (n = 573)	PCs in CMHC (n = 950)	Total PCs (n = 3174)
**Clinical reason for consultation, n (%)**				
Self-harm/Suicide attempt	428 (26%) *	104 (18%)	132 (14%)	665 (21%)
Acute psychosis	174 (11%)	32 (6%)	128 (13%) *	334 (11%)
Depressive symptoms	152 (9%)	95 (17%)	211 (22%) *	459 (14%)
Acute anxiety state	243 (15%)	15 (3%)	191 (20%) *	450 (14%)
Aggressiveness/psychomotor agitation	237 (14%) *	44 (8%)	60 (6%)	341 (11%)
Socio-environmental emergency	31 (2%)	0	19 (2%)	50 (2%)
Substance/medication intoxication or withdrawal	238 (14%) *	46 (8%)	28 (3%)	313 (10%)
Psychiatric symptoms due to medical disorders	44 (3%)	93 (16%) *	6 (1%)	143 (5%)
Mania	34 (2%)	6 (1%)	40 (4%)	80 (3%)
Insomnia	18 (1%)	15 (3%)	48 (5%) *	81 (3%)
Psycho-pharmacotherapy monitoring	8 (0%)	23 (4%) *	3 (0%)	34 (1%)
Eating disorders	9 (1%)	44 (8%) *	0 (0%)	53 (2%)
Medication side effects	6 (0%)	14 (2%) *	1 (0%)	21 (1%)
Others	21 (1%)	40 (7%) *	83 (9%) *	144 (5%)

Statistical analysis: Pearson’s χ^2^ test. Reason for consultation: χ^2^ = 951.90, *p* < 0.001. * Standardized Residuals (SR) ≥ 2, *p* < 0.05.

**Table 4:** Clinical interventions performed during PCs in ER, GH and CMHC.

Variables	PCs in ER (n = 1651)	PCs in GH (n = 573)	PCs in CMHC (n = 950)	Total PCs (n = 3174)
**Interventions during consultation, n (%)**				
Individual/family interview	556 (34%) *	153 (27%)	278 (29%)	987 (31%)
Compulsory health assessment **	12 (1%)	0	30 (3%) *	42 (1%)
Patient cannot be interviewed	2 (0%)	1 (0%)	0	3 (0%)
Interview + medication administration	356 (22%) *	9 (2%)	132 (16%)	497 (16%)
Interview + prescription home therapy	479 (29%)	308 (54%) *	456 (39%) *	1243 (39%)
Interview + request for laboratory tests/non-psychiatric consultation	54 (3%)	25 (4%) *	15 (3%)	94 (3%)
Interview + medication administration + therapy prescription	115 (7%) *	2 (0%)	25 (4%)	142 (4%)
More than one intervention	67 (4%)	68 (12%) *	14 (5%)	149 (5%)

Statistical analysis: Pearson’s χ^2^ test. Interventions during consultation: χ^2^ = 399.01, *p* < 0.001. * Standardize Residuals (SR) ≥ 2, *p* < 0.05. ** According to Laws 180/78 and 833/78.

**Table 5:** Outcomes of PCs in ER, GH and CMHC.

Variables	PCs in ER (n = 1651)	PCs in GH (n = 573)	PCs in CMHC (n = 950)	Total PCs (n = 3174)
**PC outcomes, n (%)**				
Discharge home	108 (7%) *	15 (3%)	8 (1%)	131 (4%)
Referral to CMHC/other community services	832 (51%)	89 (16%)	661 (70%) *	1582 (50%)
Private specialists	53 (3%) *	6 (1%)	5 (1%)	64 (2%)
Social Services	22 (1%) *	4 (1%)	0	26 (1%)
Psychiatric re-evaluation during hospitalization	120 (7%)	395 (69%) *	96 (10%)	611 (19%)
Compulsory health assessment **	10 (1%)	0	29 (3%) *	39 (1%)
Voluntary hospitalization	294 (18%) *	42 (7%)	60 (6%)	396 (13%)
Involuntary hospitalization **	42 (3%)	4 (1%)	21 (2%)	67 (2%)
Transfer to non-psychiatric ward	67 (4%) *	4 (1%)	0	71 (2%)
Prison/community placement	53 (3%) *	4 (1%)	3 (0%)	60 (2%)
Scheduled hospitalization	31 (2%)	5 (1%)	50 (5%) *	86 (3%)
Referral to a non-psychiatric specialist	11 (1%)	2 (0%)	11 (1%)	24 (1%)
Out-of-hospital involuntary treatment **	0	0	6 (1%) *	6 (0%)

Statistical analysis: Pearson’s χ^2^ test. PC outcomes: χ^2^ = 1500.00, *p* < 0.001. * Standardized Residuals (SR) ≥ 2, *p* < 0.05. ** According to Laws 180/78 and 833/78.

**Table 6:** Outcome of PCs in ER, GH, and CMHC by clinical reason.

Variables	Discharge Home	Referral to CMHC/Other Community Services	Voluntary Hospitalization	Involuntary Hospitalization **	Others
**Clinical reason, n (%)**					
Self-harm risk/suicide attempt	27 (16%)	362 (16%)	178 * (45%)	8 (12%)	89 (36%)
Acute psychosis	17 (10%)	167 (7%)	92 * (23%)	33 * (49%)	24 (10%)
Depressive symptoms	23 (14%)	393 * (17%)	19 (5%)	0 (0%)	24 (10%)
Acute anxiety state	35 * (21%)	392 * (17%)	8 (2%)	1 (1%)	14 (6%)
Aggressiveness/psychomotor agitation	20 (12%)	239 (10%)	31 (8%)	17 * (25%)	33 (13%)
Socio-environmental emergency	3 (2%)	40 (2%)	4 (1%)	0 (0%)	3 (1%)
Substance/medication intoxication or withdrawal	11 (6%)	235 (10%)	44 (11%)	1 (1%)	22 (9%)
Psychiatric symptoms in medical disorders	18 * (11%)	104 (5%)	2 (1%)	0 (0%)	19 * (8%)
Mania	2 (1%)	54 (2%)	13 (3%)	7 * (10%)	4 (2%)
Insomnia	7 (4%)	129 * (6%)	1 (0%)	0 (0%)	7 (3%)
Psycho-pharmacotherapy monitoring	5 (3%)	74 * (3%)	0 (0%)	0 (0%)	2 (1%)
Eating disorders	2 (1%)	28 (1%)	3 (1%)	0 (0%)	1 (0%)
Medication side effects	0 (0%)	49 * (2%)	1 (0%)	0 (0%)	3 (1%)
Others	0 (0%)	19 (1%)	0 (0%)	0 (0%)	2 (1%)

Statistical analysis: Pearson’s χ^2^ test. Clinical reason for outcome: χ^2^ = 629.958, *p* < 0.001. * Standardized Residuals (SR) ≥ 2, *p* < 0.05. ** According to Laws 180/78 and 833/78.

**Table 7:** Clinical reasons for PCs in ER, GH, CMHC by therapeutic adherence.

Variables	Good Therapeutic Adherence	Therapeutic Discontinuation < 3 Months to 1 Year	Therapeutic Discontinuation < 1 Year	Therapeutic Discontinuation > 1 Year	Total
**Clinical reason for PCs, n (%)**					
Self-harm risk/suicide attempt	471 (22%) *	26 (16%)	11 (12%)	4 (17%)	512 (21%)
Acute psychosis	189 (9%)	53 (32%)	22 (24%) *	4 (17%)	268 (11%)
Depressive symptoms	308 (14%)	14 (8%)	17 (18%)	1 (4%)	340 (14%)
Acute anxiety state	331 (15%) *	13 (8%)	10 (11%)	3 (13%)	357 (14%)
Aggressiveness/psychomotor agitation	244 (11%)	14 (8%)	12 (13%)	3 (13%)	273 (11%)
Socio-environmental emergency	26 (1%)	3 (2%)	1 (1%)	0	30 (1%)
Substance/medication intoxication or withdrawal	157 (7%)	20 (12%) *	12 (13%)	5 (22%) *	194 (8%)
Psychiatric symptoms in medical disorder	113 (5%)	3 (2%)	3 (3%)	0	119 (5%)
Mania	61 (3%)	8 (5%)	3 (3%)	3 (13%)	75 (3%)
Insomnia	75 (3%)	1 (1%)	0	0	76 (3%)
Psychopharmacological therapy monitoring	30 (1%)	1 (1%)	2 (2%)	0	33 (1%)
Eating disorders	30 (1%)	1 (1%)	2 (2%)	0	33 (1%)
Medication side effects	20 (1%)	0	0	0	20 (1%)
Others	118 (5%)	10 (6%)	0	0	128 (5%)

Statistical analysis: Pearson’s χ^2^ test. Clinical reason for PCs by treatment adherence: χ^2^ = 164.19, *p* < 0.001. * Standardized Residuals (SR) ≥ 2, *p* < 0.05.

**Table 8:** Outcomes of PCS in ER, GH and CMCH by therapeutic adherence.

Variables	Good Therapeutic Adherence	Therapeutic Discontinuation < 3 Months to 1 Year	Therapeutic Discontinuation < 1 Year	Therapeutic Discontinuation > 1 Year	Total
**PC outcome, n (%)**					
Discharge home	62 (3%) *	0	0	0	62 (3%)
Referral to CMHC/other community services	1086 (50%)	90 (54%)	49 (53%)	13 (57%)	1238 (50%)
Private specialist	36 (2%)	2 (1%)	0	0	38 (2%)
Social Services	10 (0%)	1 (1%)	1 (1%)	0	12 (0%)
Psychiatric re-evaluation during hospitalization	468 (21%) *	22 (13%)	7 (8%)	1 (4%)	498 (20%)
Compulsory health assessment **	15 (1%)	15 (9%) *	1 (1%)	0	31 (1%)
Voluntary hospitalization	285 (13%)	17 (10%)	25 (27%) *	4 (17%)	331 (13%)
Involuntary hospitalization **	25 (1%)	11 (7%) *	6 (6%) *	4 (17%) *	46 (2%)
Transfer to no-psychiatric ward	47 (2%)	2 (1%)	0	0	49 (2%)
Prison/community placement	53 (2%)	2 (1%)	0	0	55 (2%)
Scheduled hospitalization	80 (4%) *	1 (1%)	1 (1%)	0	82 (3%)
Referral to a non-psychiatric specialist	15 (1%)	0	1 (1%)	1 (4%) *	17 (1%)
Out-of-hospital involuntary treatment **	0	4 (2%) *	2 (2%) *	0	6 (0%)

Statistical analysis: Pearson’s χ^2^ test. PC outcomes by treatment adherence: χ^2^ = 263.97, *p* < 0.001. * Standardized Residuals (SR) ≥ 2, *p* < 0.05. ** According to Laws 180/78 and 833/78.

**Table 9:** Multivariate Logistic Regression: variables statistically significantly associated with hospitalization (Hospitalization = 1; No Hospitalization = 0).

Variables	Odds Ratio	95% CI	*p*-Value
**Clinical setting of PC**			
CMHC	0.31	0.21 to 0.43	<0.001
**Substance use**			
Polysubstance use	2.28	1.34 to 3.90	0.002
**Therapeutic adherence**			
Therapeutic discontinuation < 1 year	2.05	1.02 to 4.13	0.043
Therapeutic discontinuation > 1 year	8.00	2.27 to 28.24	0.001
**Clinical reasons for PC**			
Acute psychosis	1.66	1.03 to 2.66	0.036
Depressive symptoms	0.21	0.11 to 0.39	<0.001
Acute anxiety state	0.11	0.05 to 0.23	<0.001
Socio-environmental emergency	0.17	0.035 to 0.86	0.032
Substance/medication intoxication or withdrawal	0.26	0.14 to 0.50	<0.001
Psychiatric symptoms in medical disorder	0.28	0.09 to 0.90	0.032
Psychopharmacological treatment monitoring	0.13	0.025 to 0.69	0.016
Aggressive-ness/psychomotor agitation	0.34	0.20 to 0.59	<0.001
**PC interventions**			
Interview + medication administration	0.35	0.23 to 0.53	<0.001
Interview + prescription home therapy	0.041	0.023 to 0.071	<0.001
Interview + request for laboratory tests and/or non-psychiatric consultation	0.27	0.11 to 0.64	0.003
Interview + medication administration + prescription home therapy	0.18	0.07 to 0.42	<0.001

Abbreviations: CMHC, Community Mental Health Center; CI, confidence interval. Statistical significance was set at *p* < 0.05.

## Data Availability

The original contributions presented in this study are included in the article; further inquiries can be directed to the corresponding author, but due to privacy and ethical restrictions, individual data are unavailable.

## Abbreviations

The following abbreviations are used in this manuscript:

PC	Psychiatric Consultation
CMHC	Community Mental Health Center
ER	Emergency Room
GH	General Hospital
CL	Consultation-Liaison
SPDC	Service for Psychiatric Diagnosis and Care
LAI	Long-Acting Injection
